# Throwing a spotlight on genomic dark matter: The power and potential of transposon-insertion sequencing

**DOI:** 10.1016/j.jbc.2025.110231

**Published:** 2025-05-14

**Authors:** Laura M. Nolan, Mark A. Webber, Alain Filloux

**Affiliations:** 1 Singapore Centre for Environmental Life Sciences Engineering, Nanyang Technological University, Singapore; 2 School of Biological Sciences, Nanyang Technological University, Singapore; 3 Quadram Institute Bioscience, Norwich Research Park, Norwich, UK; 4 Norwich Medical School, Norwich Research Park, Norwich, UK; 5 Center for Microbial Interactions, Norwich Research Park, Norwich, UK; 6 Lee Kon Chian School of Medicine, Nanyang Technological University, Singapore; 7 Imperial, Centre for Bacterial Resistance Biology, London, UK

**Keywords:** transposon mutagenesis, TraDIS, Tn-seq, bacterial genomics, high-throughput screening, linking genotype-phenotype, novel genes, dark matter

## Abstract

Linking genotype to phenotype is a central goal in biology. In the microbiological field, transposon mutagenesis is a technique that has been widely used since the 1970s to facilitate this connection. The development of modern 'omics approaches and next-generation sequencing have allowed high-throughput association between genes and their putative function. In 2009, four different variations in modern transposon-insertion sequencing (TIS) approaches were published, being referred to as transposon-directed insertion-site sequencing (TraDIS), transposon sequencing (Tn-seq), insertion sequencing (INSeq), and high-throughput insertion tracking by deep sequencing (HITS). These approaches exploit a similar concept to allow estimation of the essentiality or contribution to fitness of each gene in any bacterial genome. The main rationale is to perform a comparative analysis of the abundance of specific transposon mutants under one or more selective conditions. The approaches themselves only vary in the transposon used for mutagenesis, and in the methodology used for sequencing library preparation. In this review, we discuss how TIS approaches have been used to facilitate a major shift in our fundamental understanding of bacterial biology in a range of areas. We focus on several aspects including pathogenesis, biofilm development, polymicrobial interactions in various ecosystems, and antimicrobial resistance. These studies have provided new insight into bacterial physiology and revealed predicted functions for hundreds of genes previously representing genomic “dark matter.” We also discuss how TIS approaches have been used to understand complex bacterial systems and interactions and how future developments of TIS could continue to accelerate and enrich our understanding of bacterial biology.

## Background

The advent of genomics has led to the exponential production of bacterial genome sequences, since the first description of the *Haemophilus influenzae* genome in 1995 (1). Of this multitude of gene sequences, a large proportion, 40 to 60% in each genome, has no assigned function (2). As of today, there are more than 2 million assembled bacterial genomes available (3). Our ability to continue generating sequence information has increased hugely since the development and widespread use of next-generation sequencing platforms, with the cost of generating a megabase of DNA sequence dropping to fractions of a dollar in the last 15 years. These sequences are from thousands of different species and continue to increase as more sequences are continuously being generated. These genome sequences reveal predicted genes and corresponding amino acid sequences of hundreds of millions of proteins. Only a small proportion of these proteins have been experimentally studied, with the function of most being predicted from their similarity to experimentally characterized proteins. This is further complicated by the fact that approximately one-third of bacterial proteins are not similar enough to any characterized protein and so their function cannot be predicted *via* this method (2). This means that there are vast amounts of genomic “dark matter”, *i.e.* genes which encode for hypothetical proteins, with the gap from gene sequence to protein function being a major challenge for biology. Given the surfeit of genomic information that is available, new methods to determine the functions of large numbers of genes are required. Various approaches can be taken to achieve this, for example, transcriptomics, genome-wide association studies (GWAS), and generation of defined mutants. While all these approaches can increase understanding of the functional roles of many genes, the outputs can be limited when there is no existing annotation for a gene.

Transposon mutagenesis became a widely used genetic method in the 1990s to predict the function of genes. One such method is signature-tagged mutagenesis (STM), which was used in a variety of studies, including those to define virulence genes, for example identifying pathogenicity factors in *Salmonella* Typhimurium (4). In many cases these studies allowed the field to move from predicting the biological role of gene products to assigning functional roles using empirical evidence. Another example of the early use of transposon mutagenesis was in biofilm research where transposon mutant libraries allowed identification of the first key molecular determinants of bacterial attachment to a surface, prior to biofilm formation (5, 6).

Transposon mutagenesis works by a transposon inserting into the bacterial chromosome, which can result in a phenotypic impact or fitness cost, that can then be used to link this biological function to a specific gene. This approach benefits from not requiring prior knowledge about the target genome, and wide applicability to many bacterial species. Whilst there is some insertion bias for most transposons, as long as sufficient insertion density is obtained this will not impact the ability of TIS to link genotype-phenotype (see Opijnen *et al.*, (7) for a detailed discussion on these aspects including insertion bias and transposon selection). The limitation of classic transposon mutagenesis approaches is that they are relatively labor-intensive and low throughput. These limitations have been overcome by transposon insertion sequencing (TIS) approaches that link the generation of very large and highly saturated transposon mutant libraries with high throughput sequencing, allowing genomic “dark matter” to now be addressed on a large scale. Due to the high number of transposon insertions, TIS approaches allow statistical tests to be performed on the relative frequency of transposon insertions in genes between different conditions/libraries, this allows statistical assessments of the significance of all genes in the genome for the phenotype being studied to be made in parallel. In this article, we briefly review the history of TIS methods, describe some important findings from TIS studies to date, and provide future perspectives on new ways this methodology either has been used, or could be used, in combination with other cutting-edge approaches.

## Transposon-insertion sequencing (TIS) approaches

**Figure 1:**
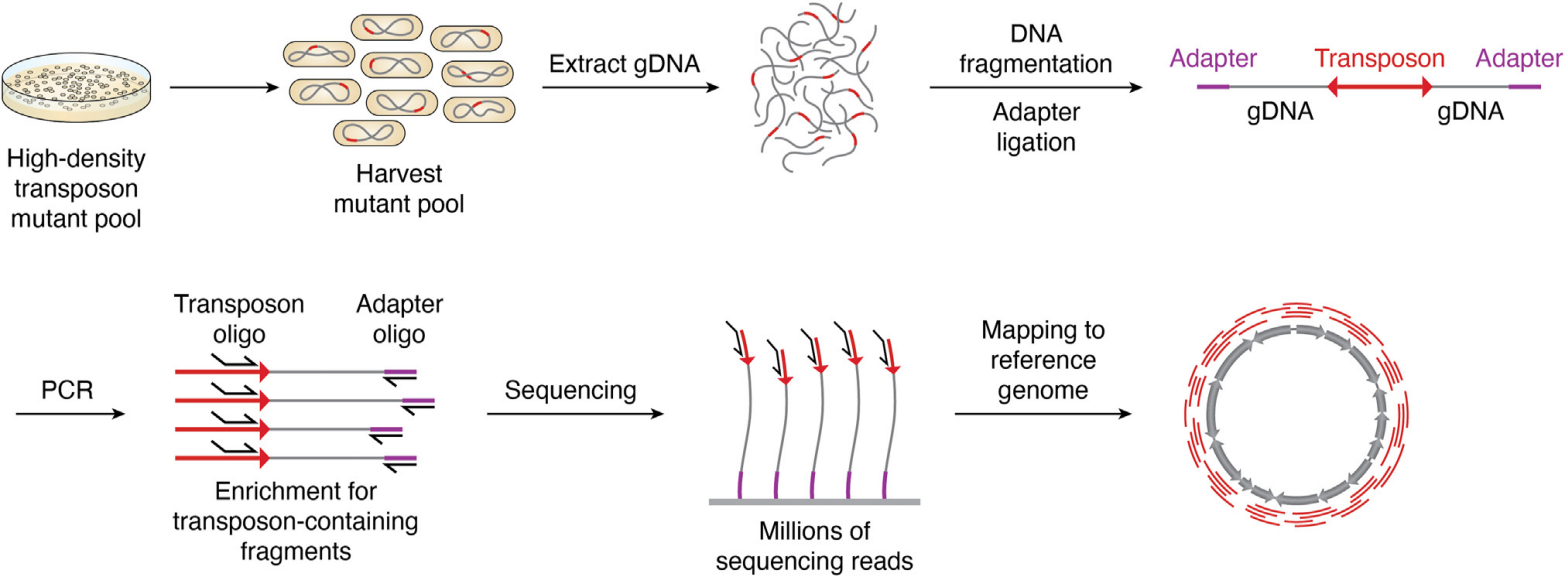
**Overview of TIS library sequencing.** A high-density transposon mutant pool is generated *via* transformation of a transposon into cells, where it is then inserted at random into the target genome. The pool of mutants is harvested, and the genomic DNA is extracted. The DNA is then fragmented, either by shearing or enzymatic treatment (depending upon the kind of TIS being performed), and adapters ligated at the 5′ and 3′ end of the fragment. A PCR step is performed with transposon and adapter-specific oligos to enrich for transposon-containing fragments. Sequencing is then performed outwards from the transposon, with reads being mapped back to the reference genome to determine the location of all sites of transposon insertion.

In 2009, four conceptually similar transposon-insertion sequencing (TIS) methods were developed in parallel, Tn-seq, TraDIS, INSeq, and HITS. These have been reviewed in detail elsewhere, along with descriptions of the features that distinguish each method (7, 8). Each has a similar core rationale where transposons (typically carrying an antibiotic resistance cassette to select for transformants) are introduced by electroporation or conjugation into a target genome (the transposons do not contain a transposase sequence of their own, so are immobile once introduced) before mutants are recovered and pooled. The most common transposons used in TIS are Tn*5* and *mariner*, and these are delivered into cells either on a non-replicative plasmid or *via* a transposome (9). At this point, the TIS library is often characterized, *i.e.* transposon insertion sites for all mutants are amplified and sequenced in parallel, then mapped back to the reference genome (see Fig. 1). This library is typically referred to as the base or input library and can be used to determine whether there is a sufficient density of transposon insertions and a lack of insertion bias across the genome. Additionally, genes lacking insertions in this base/input library are usually identified and represent essential genes under the conditions in which the library was constructed. The TIS library is then grown under conditions of interest (*e.g.* ± nutrient, ± antibiotic) or used in an assay of interest (*e.g.* passaged through a mouse or inoculated into a biofilm). The pool of mutants (often called the output library) capable of surviving the experimental conditions is obtained, and their DNA is extracted and sequenced in the same manner as the base/input library. The transposon insertion sites and frequency of insertion in the mutant pools are then compared to identify conditionally-important genes required for the phenotype of interest, *e.g.* comparing mutants recovered from growth in the presence vs. absence of an antibiotic reveals genes required for growth in the presence of the antibiotic, or comparing the mutants recovered from a mouse model *versus* the base/input mutant pool that was inoculated reveals genes required for *in vivo* colonization/survival. For more detailed discussion on TIS library preparation and pipelines for data analysis, the reader is referred to several excellent publications (8, 10, 11).

The use of TIS approaches has allowed scientists to predict functions of genes *en masse*, providing a solid foundation for additional experimental work to further advance our biological understanding in many areas. In this review we will look in more detail at some key examples of how TIS approaches have been used in four broad research areas: (i) antimicrobial resistance (AMR) mechanisms and antimicrobial drug discovery; (ii) biofilm development and community interactions in biofilms; (iii) bacterial virulence, infection strategies and host-pathogen interactions; and (iv) bacterial physiology and the “rules of life”.

## AMR mechanisms and antimicrobial drug discovery

Bacteria demonstrating AMR are continuing to be isolated more frequently across the globe and AMR has been identified as one of the great challenges for humanity in the coming decades (12). There are many challenges that need to be addressed to tackle this issue, including characterizing antibiotic modes of action and resistance mechanisms, as well as finding new antibacterial therapeutics that effectively target bacterial pathogens. TIS approaches have been used to address all these challenges in diverse species, as discussed below.

Understanding what makes a bacterial cell susceptible or resistant to an antibiotic can reveal information about the resistance mechanisms but can also provide clues as to the mode of action of the antibiotic. Several groups have used TIS to study susceptibility and resistance mechanisms to important antibiotics in pathogenic bacteria of clinical importance. Xu *et al.* (13) used TIS to study antibiotic resistance mechanisms of *Mycobacterium tuberculosis*, the causative agent of tuberculosis (TB) and the leading global cause of death from infection (14). Treating TB is challenging due to the slow growth rate of *M. tuberculosis,* with the standard treatment regimen taking at least 6 months when the infecting strains are drug sensitive. In complicated cases of multidrug-resistant (MDR) and extensively drug-resistant (XDR) *M. tuberculosis*, the additional use of second-line drugs is required for 18 months or more. Here, the authors used TIS in a chemical genetic interaction (CGI) profiling approach to identify genes involved in determining susceptibility to partially-inhibitory concentrations (*i.e.* below the minimum inhibitory concentration (MIC)) of five different antibiotics (rifampicin, ethambutol, isoniazid, vancomycin, and meropenem), which all have diverse mechanisms of action (13). CGI profiling quantifies the susceptibility of a defined set of mutants to a set of chemical compounds and uses machine learning (ML) to predict the mechanism of action. Determinants affecting resistance to more than one antibiotic were identified, and this revealed the cell-envelope to be important for all the drugs. Specific genes involved included those encoding the protein translocase SecA2, mannosyltransferase PimE, peptidoglycan-arabinogalactan ligase Lcp1, mycolic acid synthase MmaA4, cell envelope-associated protease CaeA/Hip1, and FecB, which is a putative iron dicitrate-binding protein (13). Lcp1, MmaA4 and PimE, all have a direct role in controlling cell envelope biogenesis, homeostasis, and therefore cell envelope integrity and permeability (15, 16, 17, 18). SecA2 is known to export virulence factors that are required for pathogenesis, rather than antimicrobial resistance, although factors transported by SecA2 could also be required for cell envelope integrity, hence the link with resistance (19). Interestingly, FecB is predicted to be involved in uptake of iron and heme. Yet the present study suggests a novel role in contributing to resistance to all antibiotics by controlling cell envelope permeability. This was validated by measuring accumulation of ethidium bromide which was higher in the *fecB* mutant (13). This work has documented novel roles for several genes in impacting activity of multiple antibiotics, directly or indirectly, as well as identifying specific cellular components that represent high-value targets relevant to many antibiotics. Development of inhibitors of these targets could potentiate existing antibiotic regimes and/or increase susceptibility of *M. tuberculosis* to antibiotics that are normally ineffective against this bacterium. Of the genes identified in this study, 77 were in the genomic “dark matter” category (Table 1), which now may be assigned a predicted role in *M. tuberculosis* resistance to one or more clinically relevant antibiotics (13).

**Table 1:** Number of “dark matter” genes identified from TIS outputs

Publication	Bacterial species	Phenotype of interest	Mutants selected by	Number of genes^a^
Xu *et al.,* 2017	*M. tuberculosis*	Antibiotic (rifampicin, ethambutol, isoniazid, vancomycin, meropenem) susceptibility	Present in input library/absent with sub-MIC antibiotic	77
Co *et al.,* 2019	*S. aureus*	Antibiotic (daptomycin) susceptibility	Present in input library/absent with sub-MIC antibiotic	21
Boinett *et al.,* 2019	*A. baumannii*	Antibiotic (colistin) susceptibility	Present in input library/absent with sub-MIC antibiotic	6
Turner *et al.,* 2020	*E. coli*	Antibiotic (fosfomycin) susceptibility	Present in input library/absent with sub-MIC antibiotic	0
Nolan *et al.,* 2021	*P. aeruginosa*	T6SS (toxin)-immunity genes	Present in T6SS inactive library/absent in T6SS active library	7
Schinner *et al.,* 2020	*P. aeruginosa*	Biofilm development	Present in input library/absent in biofilm biomass	11
Nolan *et al.,* 2018	*P. aeruginosa*	Active biofilm expansion	Present in input library/absent in expanded biofilm	98
Holden *et al.,* 2021	*E. coli*	Biofilm development	Present in input library/absent in biofilm biomass	5
DeFrancesco *et al.,* 2017	*S. aureus*	Mechanism of eDNA release in biofilms	Present in gDNA/absent in eDNA	1
Ibberson *et al.,* 2017	*S. aureus* and *A. actinomycetemcomitans*	Essential genes for co-infection with *P. aeruginosa* or *S. gordonii*	Present in input library/absent from co-infection	1680
Warr *et al.,* 2019	*E. coli* (EHEC)	*In vivo* growth	Present in input library/absent from infant rabbit colon passage	210
Breton *et al.,* 2017	*S. pyogenes,* GAS	*In vivo* growth	Present in input library/absent from murine skin wound passage	54
Price *et al.,* 2018	32 different bacteria	Growth under diverse conditions	Present in input library/absent from tested condition	>10,000
Duncan *et al.,* 2018	*V. cholerae*	Mechanisms of *Bdellovibrio* predation	Present in input library/absent after inoculation with *Bdellovibrio*	21
Hardy *et al.,* 2021	*L. pneumophila*	Natural transformation and competence induction	Present in input library/unable to uptake DNA conferring resistance	13

a Refers to gene being a hypothetical and within cut off criteria for the study.

Infection by antibiotic-resistant *Staphylococcus aureus*, a member of the ESKAPE group of pathogens, is another major clinical issue due to large numbers of infections and limited treatment options (20). The major antibiotic class that has historically been used to treat *S. aureus* infections are the *β*-lactams. However, treatment efficacy has been challenged by the emergence of methicillin-resistant strains (MRSA) (20). In these cases alternative antibiotics such as daptomycin are used (20) and while most *S. aureus* strains remain susceptible, isolates resistant to this antibiotic have been reported with increasing frequency (21). Coe *et al.* (22) used TIS to identify genes and pathways important for resistance to sub-inhibitory concentrations of daptomycin of five different *S. aureus* strains from three important clonal complexes (HG003, USA300-TCH1516, MSSA476, MW2, and MRSA252). The aim was to identify universally conserved daptomycin resistance factors that could highlight a way forward to combat resistance to this antibiotic in *S. aureus*. This resulted in the identification of several genes/pathways that were predicted to be involved in daptomycin susceptibility, and as for *M. tuberculosis*, they were mostly related to the cell envelope. This included the lipoteichoic acid (LTA) biosynthesis pathway. LTA is a polymer anchored in the cell wall, and modifications to LTA have been shown to be key for resistance to antibiotics and cationic antimicrobial peptides (AMPs) (23, 24). Genes identified using the TIS approach by Coe *et al.* (22) encoded LtaA, a flippase that translocates the LTA precursor across the cytoplasmic membrane, as well as enzymes GtaB, PgcA and UgtP, required for the synthesis of the LTA precursor, Glc_2_DAG (22). Other genes encoding enzymes involved in cell wall synthesis and/or membrane synthesis were also identified (MurA1, Alr1, and MprF) as well as several components and/or signaling systems that are involved in regulating processes in the cell envelope (GraRS/VraFG, ArlR, GpsB, EzrA, and Noc). Several of these components had previously been linked to daptomycin resistance but not all, notably LtaA and GpsB. GpsB has been proposed to have a role in cell division and cell morphology but has also been shown to interact with a protein, TarG, which is required for wall teichoic acid (WTA) transport (25). While LTAs are anchored in the membrane, WTAs are anchored in the cell wall but both contribute to cell envelope integrity. This study suggests that targeting these cell envelopes associated components and/or pathways could be used to resensitize daptomycin-resistant *S. aureus* isolates to this antibiotic. In addition to identifying genes with known or putative roles, there were also 21 genomic “dark matter” genes identified (Table 1), which are now predicted to have a role in daptomycin resistance (22).

Treatment of *Acinetobacter baumannii* infections is another common clinical challenge due to rising numbers of MDR strains and the ability of this bacterium to acquire and accumulate resistance genes (26). Some strains have acquired genomic regions encompassing *ca* 100kb and containing up to 45 antimicrobial resistance genes (27). Boinett *et al.* (28) study resistance of *A. baumannii* to colistin. Colistin is an older generation last-line antimicrobial that is often used alone or in combination with tigecycline, carbapenems or rifampicin however, even with the use of combinatorial treatment regimes, hetero-resistance and complete resistance to colistin is still frequently reported (29). Here TIS was performed on a colistin-sensitive *A. baumannii* clinical isolate which was subjected, in two passaging steps, to a sub-inhibitory concentration of colistin. Genes required for colistin survival included those involved in synthesis of cell envelope components, namely lipo-oligosaccharide (LOS) (*lpxO, lpsC,* and *mfpsA*) and peptidoglycan (*mcrB* and *galE*). In addition, *sigX*, which belongs to the family of extra-cytoplasmic function (ECF) sigma factors involved in cell envelope stress response in several bacteria, including *Pseudomonas aeruginosa* (30) and *Bacillus subtilis* (31), was found to be involved in survival of *A. baumannii* to colistin. Genes in the *mla* locus (*mlaC, mlaF*, and *mlaD*), which are involved in maintaining cell surface lipid symmetry and outer membrane stability (32, 33) were also found to be required for colistin survival. Genes required for susceptibility to colistin included putative pilus assembly genes (28). A previous study reported a decrease in outer membrane structures, including pili, when there is disruption or damage to the outer membrane, as it is thought that loss of these structures reduces membrane “gaps” and helps to maintain cellular integrity (34). Together these data highlight that maintenance of lipid homeostasis, as well as stability of the cell envelope, is crucial for *A. baumannii* to survive colistin exposure (28). This suggests that the modifications *A. baumannii* make to its cell envelope in response to colistin (28) limit colistin’s ability to access or target LPS at the inner membrane. In this study, six genes fell into the genomic “dark matter” category, which now have a predicted role in either colistin tolerance or susceptibility (Table 1).

This study by Boinett *et al.* (28) adds to our understanding of the mode of action of colistin as well as how *Acinetobacter* develops resistance to this antibiotic. Many other important pathogens are resistant to colistin (35), and the resistance mechanisms identified in this study could provide a foundation for understanding colistin resistance in a range of bacteria. From this and the previous studies presented so far in this review, it is increasingly evident that the composition and integrity of the bacterial cell envelope is one major factor that controls and dictates antibiotic resistance and/or susceptibility. These studies suggest that many of these cell envelope targets should be considered for drug development and show that many potentially high-value targets may be species-specific. In some cases, potential drug targets are regulatory systems that respond to membrane stress, in other cases, components involved in the biogenesis of lipids, LPS, or peptidoglycan. Although targeting these physiological processes is not particularly novel, TIS approaches can precisely identify the most promising component(s) within these processes for inhibition. This can guide the development of innovative inhibitors of novel components of established targets, as has been successfully shown with the advent of teixobactin targeting lipid precursors of peptidoglycan and cell wall teichoic acid (36).

While many antibiotics attack the cell envelope, as in the examples above, transport across the cell envelope is often required for efficacy. We used TIS to identify the genetic determinants in *Escherichia coli* involved in susceptibility to fosfomycin, which has been used increasingly to treat multidrug-resistant infections (37). Genes were identified that were involved in determining susceptibility to fosfomycin across a range of concentrations from 0.25 times to 2 times the MIC. These included *murA,* which encodes the enzyme involved in the first step of peptidoglycan synthesis and the known target of fosfomycin (38). Another gene hit, *glpT,* encodes one of the two nutrient importer systems known to facilitate entry of fosfomycin into the cell (39). We also identified *cyaA* encoding an adenylcyclase, and *ptsI,* encoding a phosphotransferase, to be involved in susceptibility. Both CyaA and PtsI control intracellular levels of cyclic AMP that in turn, regulate expression of *glpT* (40). Genes involved in susceptibility that had not been previously identified included multiple genes in the *phn* operon, which encodes components involved in phosphonate uptake and degradation, as well as an ABC phosphate importer, PstSACB. This indicates that fosfomycin may also be able to use this importer as a route of entry into the cell (37). Interestingly, *mutL* and *tatD,* which encode a DNA mismatch repair protein and a DNA repair exonuclease, respectively, were beneficial for growth in the presence of fosfomycin. This suggests that fosfomycin causes some DNA damage, likely due to altered metabolism in response to fosfomycin-mediated inhibition of its primary target, MurA, as has been shown to occur when cells are treated with bactericidal antibiotics (37, 41). None of the genes from the TIS output in this study fell into the “dark matter” category, *i.e.,* all genes already had some predicted functions. This is unsurprising given the fact that *E. coli* has been studied for decades since its discovery in 1885 and its advent as a preferred laboratory model organism. However, the power of TIS is again demonstrated as we were able to reassess and refine functions for several genes.

**Figure 2:**
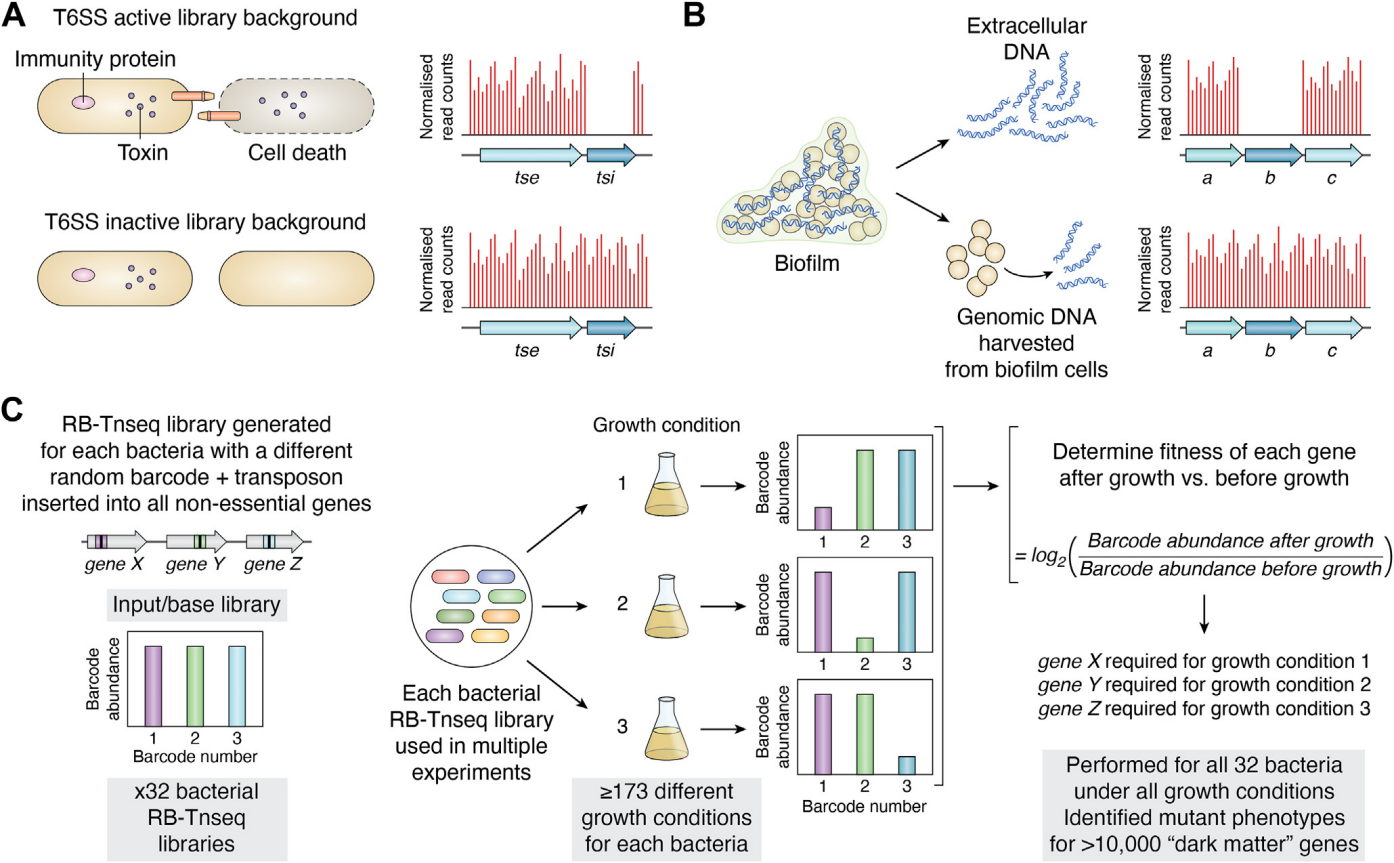
**Examples of using TIS to increase biological understanding.***A*, Using TIS to identify *P. aeruginosa* T6SS immunity genes (*tsi*) and adjacent toxin genes (*tse*) (44). Two high-density TIS libraries were generated in a T6SS active or inactive background. Each pool of mutants was separately incubated at high-density to promote cell contact-dependent T6SS killing *via* delivery of toxins into cells lacking the cognate immunity. This delivery only occurs in a T6SS active background which, results in cell death and absence of the *tsi* mutant in the library pool. Transposon insertions (*red* lines in graphs) are tolerated in the cognate immunity gene (*tsi*) only in the T6SS inactive background. *B*, identification of genes involved in *S. aureus* eDNA release in biofilms (78). A TIS library of *S. aureus* was used to grow biofilm. Extracellular DNA (eDNA) was separated from the genomic DNA of biofilm-grown cells and sites of transposon insertion in each pool identified. Genes involved in eDNA release are absent in the eDNA pool, and thus transposon insertions are not observed in these genes (*e.g. gene b* in the right-hand graph has no transposon insertions, as represented by the *red* lines in the graph) but present in all genes in the genomic DNA pool. *C*, assigning phenotypes for genes in diverse bacteria under many conditions using RB-Tnseq (113). For all 32 bacteria in the study a random-barcode Tn-seq (RB-Tnseq) library was generated, whereby a unique barcode (*colored* box) is associated with the transposon (*black* box) and randomly inserted into the genome, which creates a unique barcode for each transposon insertion site. Here shown are unique barcode-transposons in 3 genes, *gene X, Y*, and *Z.* The input library is sequenced to identify the gene that each unique barcode transposon is inserted into. The pooled RB-Tnseq library can then be inoculated into multiple selective conditions. In the Price *et al.* (113) study there were at least 173 conditions assayed for each bacterium. After growth a PCR step is performed followed by sequencing to determine the barcode abundance, which can then be used to determine the fitness of each gene under the conditions assayed. This can include significant numbers of “dark matter” genes as was the case in this study by Price *et al.* (113).

The abovementioned studies demonstrate how TIS can systematically identify genes involved in conferring resistance or susceptibility to antibiotics in diverse bacterial species. In addition to understanding these aspects there have been significant efforts made to identify antimicrobials with novel mechanisms of action. One avenue to identify such potential novel antimicrobial therapies is to study the mechanisms bacteria have evolved to compete with one another over billions of years. Many bacteria inhibit growth or survival of their neighbors using an array of different mechanisms from contact-dependent toxin/inhibitor delivery to production of diffusible toxins and growth inhibitors. The cellular targets of these competitive strategies have been effectively validated over the course of evolution and thus point to potentially effective ways to target key components of bacterial cells. The type VI secretion system (T6SS) is a nano-weapon used by Gram-negative bacteria for interbacterial competition. The T6SS delivers toxins into sensitive prey cells in a contact-dependent manner (42), with the producing cell and neighboring sister cells protected from self-intoxication by production of immunity proteins for each bacterial-targeted toxin (43). Our previous study aimed at identifying T6SS immunity genes and cognate toxins in *P. aeruginosa* by exploiting the fact that toxin and immunity genes are always encoded next to one another in the genome (44) (Fig. 2*A*). We generated two TIS libraries, one in a background where the T6SS was active and another in a T6SS inactive background. We reasoned that insertions in immunity genes would mean that mutants would not be recovered in the T6SS active library background, but they would be enriched in the T6SS inactive library background. By comparing the relative frequency of transposon insertion in our two libraries we were able to identify both known T6SS toxin-immunity pairs, as well as several putative novel pairs. Our screen identified a total of 42 putative immunity genes, of which seven are in the genomic “dark matter” category (Table 1), which are now predicted to have a role in protecting the cell against T6SS-mediated killing. We also performed a detailed characterization of one novel toxin, Tse8, that led to identification of a naturally validated antibacterial target, which could be used to develop novel therapeutics against (44). Here the antibacterial target was the bacterial transamidosome complex, composed of GatABC, which is essential for protein synthesis in bacteria that lack one or more of the glutamine or asparagine tRNA synthases. Many ESKAPE and high-priority pathogens rely upon the transamidosome for protein synthesis, and in the case of *M. tuberculosis* it has been proposed that the utilization of the transamidosome is favorable since it is more error-prone and facilitates adaptation to stressful environments, including antibiotic treatment (45). We found that our T6SS toxin, Tse8, is a GatA homologue and that it inhibits protein synthesis by recruiting more copies of GatA to the transamidosome complex, which overall alters the fine-tuned stoichiometry of the complex, inactivating it. Our findings could be exploited to identify or design small molecules (antibiotics) that target the transamidosome complex, either in the same or a different way to Tse8. Although tRNA synthases have previously been considered as drug targets, such as the aspartyl tRNA synthase AspS in *M. tuberculosis* (46), the transamidosome has not yet been considered. This is a proof-of-concept study that demonstrates that TIS could be used to identify non-traditional targets for antimicrobial development, which may include more T6SS toxin-immunity genes but also other toxin-immunity genes that are delivered by other mechanisms (*e.g.* Type IV, type V, and type VII secretion systems [T4SS, T5SS, and T7SS]). This could ultimately uncover an unexplored reservoir of naturally validated antibacterial targets.

TIS studies have proved hugely valuable in determining the landscape of antibiotic resistance mechanisms in important pathogens and broadened our understanding of factors that influence susceptibility of pathogens to many important antibiotics. Components within the cell envelope were commonly hit in these TIS studies. This reinforces the idea that TIS is a powerful tool to lead us to the most promising cellular components to target to inactive bacteria. This provides a solid foundation for ongoing work to develop new strategies to target multidrug-resistant pathogens. In terms of increasing our understanding of the biological function of genomic “dark matter,” the studies which included either multiple strains (22) or multiple antibiotics (13) were able to assign predicted function to the greatest number of genes that previously fell into this category. Therefore, while it is certainly informative to use TIS approaches that focus on single strain/antibiotic combinations, studies that include multiple strains/antibiotics are likely to increase knowledge of genomic “dark matter” relative to AMR. Furthermore, and given the increasing awareness of the importance of the pangenomes in many species, studies with multiple strains will be able to provide information about the importance of accessory genomes rather than just the core genome of laboratory strains. One other important consideration for studies on antibiotic resistance/sensitivity is that the effectiveness of antibiotics is typically very different between *in vitro* and *in vivo* conditions (47). Thus, whilst *in vitro* studies can shed light on how bacteria respond to antibiotics, it is important to also confirm the relevance of these responses under conditions that are relevant to the real-world setting. Given that the cost and difficulty in using TIS continues to decrease, furthering *in vivo* approaches should be a key goal of TIS studies in this area moving forward.

## Biofilm development and polymicrobial biofilm community interactions

Biofilms are communities of microbes that are encased within a self-produced extracellular matrix, they exist almost everywhere on the planet and are the major mode of growth for microbes. Biofilms can strongly influence their local environment, and this can be beneficial or detrimental depending on the circumstances (48). For instance, biofilm-associated chronic infections are very challenging to resolve using treatments such as antibiotics and disinfectants, whilst biofilms within a healthy gut microbiota are associated with many positive health outcomes; in the marine environment biofouling of ship hulls results in reduced fuel efficiency and increased emissions, whilst in industrial processes biofilms can be used as biofactories for production of many valuable commodities; and in the plant rhizosphere phytopathogens can be harmful to plant health, whilst other biofilm communities are essential for plant growth (49, 50, 51, 52). Therefore, understanding how biofilms form in different conditions is a central goal for microbiology. TIS has proven to be an important tool that has been used to understand how biofilms develop and how biofilm community member interactions impact this development.

*P. aeruginosa* is an established model organism to study biofilm development with biofilms formed by this pathogen being common in wounds, middle ear infections, the cystic fibrosis (CF) lung and in catheter-associated urinary tract infections (CAUTIs). In this section we will first focus on two studies, one by Schinner *et al.* (53), and one of our previous studies (54) that have used TIS to understand different aspects of biofilm development of this pathogen.

Schinner *et al.* (53) used TIS to determine if there were common factors that *P. aeruginosa* strains (two laboratory strains, PAO1 and PA14, and one respiratory tract isolate, ZG8038581181) use to establish mature hydrated (sessile) biofilms *in vitro* (53). The authors’ aim was to uncover core regulatory pathways and/or components that are key for biofilm development that were shared by all strains as well as to identify conserved targets that could be used to interfere with *P. aeruginosa* biofilm formation. The authors generated transposon mutant libraries in all three *P. aeruginosa* strains and used these libraries to establish mature biofilms in a static microtiter plate assay, combined with automated confocal laser scanning microscopy (CLSM). For each strain they then identified genes that were enriched or depleted in the pool of mutants capable of forming mature biofilms (*i.e.* mutants absent from the biofilm biomass are assumed to be essential for biofilm development) not compared to the base library pool and validated the biofilm phenotype of several defined mutants. They found that genes involved in the SOS response, genes encoding tRNA modifying enzymes, and genes involved in adaptation to microaerophilic growth, as well as switching between aerobic glycolysis and oxidative phosphorylation, were important for biofilm growth by all *P. aeruginosa* strains. In contrast, genes involved in motility and in quorum sensing (QS) pathways (the *Pseudomonas* quinolone signal (PQS) and LasR/I pathways) were less important for biofilm growth, which suggests that there may not be a tight link between biofilm development and quorum sensing, at least for these strains under the assay conditions. In fact, it may be that it is not the presence/absence of these systems that impacts biofilm production but rather the fine-tuned control of them to produce specific amounts of QS system molecules. This is suggested by the finding that the ratio of QS molecules *N*-(3-oxododecanoyl)-L-homoserine lactone (3OC12-HSL) and *N*-butyryl-L-HSLC4-HSL (C4-HSL) is different in broth culture vs. biofilm forming conditions, with levels of C4-HSL being higher in the latter setting (55). Another possible explanation for the lack of QS system mutants in the TIS output is that the sharing of “public goods” such as QS molecules, that occurs in biofilms, masks the phenotypic impact of a single gene mutation when that mutant is present in a pool of wildtype cells. Indeed, several previous transposon mutagenesis screens did not identify all genes known to be involved in biofilm formation (5, 6), likely due to this same phenomenon. The TIS outputs from this study found 11 genes which were in the genomic “dark matter” category that now have a predicted function in biofilm formation in at least two out of the three *P. aeruginosa* species studied.

Active biofilm expansion by *P. aeruginosa* occurs *via* type-IV pili (T4P)-mediated twitching motility and is important for establishing a mature hydrated biofilm (56). We used TIS to study active biofilm expansion *in vitro* with the aim being to identify all the components required for active biofilm expansion and increasing our understanding of how *P. aeruginosa* might use this process to establish and spread infections (54). In this study a transposon mutant library of *P. aeruginosa* strain PA14 was inoculated in a colony biofilm assay (54), which provides conditions that facilitate twitching motility-mediated biofilm expansion (57). Comparison of the transposon insertion profile of the non-twitching mutant pool (*i.e.* those mutants that did not expand beyond the inoculation point) and the twitching mutant pool (*i.e.* mutants capable of biofilm expansion away from the inoculation point) allowed identification of the full repertoire of previously identified genes required for this process in *P. aeruginosa,* as well as 39 novel genes. A subset of these were followed up revealing several novel components that are likely to have a role in T4P biogenesis and/or assembly, namely, PfpI and the ExeA-domain containing protein PA14_66580. PfpI is an intracellular protease which is predicted to be associated with the inner membrane (58). Given that PfpI is involved in controlling levels of a range of chaperones and regulatory proteins (59), it could be that the protease activity of PfpI is required to control regulators or other proteins involved in T4P biogenesis and/or assembly. Interestingly, it has been recently shown that the *pfpI* gene is among the most highly upregulated during initial attachment to a surface (60) and therefore might be important for surface expansion and initial biofilm formation. The ExeA domain of PA14_66580 suggests a link to assembly of trans-envelope nanomachines since ExeA was first characterized for transport and multimerization of the type-II secretion system (T2SS) outer membrane secretin, ExeD in *Aeromonas hydrophila* (61). Here, *PA14_66580* is encoded adjacent to the *pilMNOPQ* operon, which encodes components that link the T4P secretin, PilQ, to the inner membrane alignment subcomplex (PilMNOP), suggesting a functional link (62). Indeed, it is important to highlight that the T2SS and the T4P are nearly identical macromolecular complexes (63). A clean deletion mutant of *PA14_66580* had no significant defect in PilQ expression and/or multimerization in the outer membrane, which suggests that this gene product could instead be involved in stabilization of PilQ secretion and/or formation of other T4P components required for full pilus function (54). Functional gene enrichment analyses of the TIS outputs from this study revealed an intriguing potential link between flagella-mediated swimming motility and T4P-mediated twitching motility. Specifically, it was found that gene products encoding the flagellum and flagella components in the outer membrane and the stator, had a negative effect on twitching motility and conversely, that gene products encoding flagella anchoring and regulatory components at the inner membrane and in the cytoplasm, had a positive effect on twitching motility (54). This suggests that there could be coordinated regulation to provide a balance between expression of a functional flagellum or T4P, perhaps to facilitate the appropriate response to environmental conditions *i.e.* in liquid the flagellum is fully assembled and functional to facilitate swimming motility, and in semi-solid conditions the T4P is instead fully assembled and functional to facilitate twitching motility (54). The TIS outputs from this study predicted function for 98 genes (Table 1) that were previously in the genomic “dark matter” category (54).

*E. coli* is another model organism used to study biofilm development. We have used TIS to study how *E. coli* strain BW25113 forms biofilms *in vitro* and which gene products were important at different developmental stages (64). We used a glass bead biofilm model (65) and compared the transposon insertion profiles of mutants from planktonic growth or biofilm (early, mid, and late development time points) output pools. In total, 48 genes significantly impacted biofilm formation over time with genes encoding factors associated with type I fimbriae, curli biosynthesis and flagella-mediated motility being important at all stages of development. Adhesin production was important for the initial stages of biofilm formation, and matrix production and purine biosynthesis were important during biofilm maturation. All these components and processes have been previously implicated in biofilm development although prior work had not defined the temporal importance of each (66, 67). Characterizing when in the biofilm life cycle different processes are important provides a better understanding of the requirements within the community at different stages. For example, we identified that motility and adhesion production were important at all stages of development, rather than just for initial attachment, as has been shown previously (68). We also saw regulators whose importance varied over time, for example, *leuO*, which regulates multiple genes involved in stress responses, was highly important within early and late-stage biofilms but was dispensable between these two stages. We also identified several novel genes not previously associated with biofilm formation, which included genes involved in cell division, *zapE* and *truA,* chromosome organization (*maoP*), and five genes in the “dark matter” category (Table 1). Given that ZapE and TruA have been previously shown to be required for growth under low oxygen conditions (69) and oxidative stress conditions (70), respectively, it suggests that these gene products may also be important for growth in submerged biofilms, which also have areas of low oxygen and high oxidative stress (64). MaoP is part of a group of proteins conserved in Enterobacteria that are involved in control of chromosome conformation and segregation (71). Our study suggests that cell division and chromosome segregation in *E. coli* is also involved in biofilm development, in a yet to be determined manner. Previous work has found cell division-related genes to also be involved in biofilm development, *e.g.*, in *P. aeruginosa* in the above two biofilm studies by Schinner *et al.* (53), and our study (54), as well as in *Fusobacterium nucleatum* (72), Uropathogenic *E. coli* (UPEC) (73), *Vibrio cholerae* (74), and *Cronobacter sakazakii* (75), suggesting that factors involved in both cell division and biofilm formation is common in a range of different bacteria.

Extracellular DNA (eDNA) is a key matrix component of biofilms formed by many bacteria that has many roles in this setting including in structural integrity, protection against antibiotics and host immune system factors and horizontal gene transfer (76). eDNA in biofilms is produced by cell lysis and active secretion (76) or in the case of *P*. *aeruginosa*, *via* a dynamic process called explosive cell lysis (77). DeFrancesco *et al.* (78) used TIS to identify the factors involved in eDNA release within *S. aureus* strain HG003 biofilms using a microtiter plate biofilm assay (5) (Fig. 2*B*). They reasoned that genes involved in eDNA release would be underrepresented in the eDNA compared to the genomic DNA (gDNA). To identify the genes involved in eDNA release, they separated the cells present in the biofilm and the eDNA in the matrix by exploiting the fact that when biofilm bulk is suspended in a buffer at neutral pH (7.5), the matrix is released from the cells (79). The transposon insertion profiles of the gDNA extracted from mutants in the biofilm and the eDNA in the biofilm matrix could then be compared. This identified 36 genes that had significantly more transposon insertions in the gDNA compared to the eDNA, which encoded factors involved in nucleotide metabolism, pyruvate metabolism, glycolysis and respiration, cell wall homeostasis and cyclic-di-AMP degradation to be important for eDNA release in *S. aureus* biofilms. The authors followed up 11 of these genes, and of these, one transposon mutant (*gdpP::Tn*) and three single gene deletions (Δ*sarA,* Δ*xdrA*, and Δ*apt*) had large reductions in the amount of eDNA released as well as reduced biofilm formation and cell clumping capacity (78). GdpP is a phosphodiesterase that cleaves the second messenger cyclic-di-AMP (c-di-AMP) and, when deleted, has been shown to increase intracellular c-di-AMP levels and levels of cross-linked cell wall peptidoglycan (80). It has also been shown that a drop in cyclic-di-AMP levels *via* GdpP triggers biofilm formation in *S. aureus* (81). This suggests that this second messenger might control the switch between planktonic and biofilm lifestyles in the opposite manner to a different second messenger, cyclic-di-GMP (c-di-GMP), in Gram-negative bacteria where high cyclic-di-GMP levels promote the switch from planktonic to a biofilm lifestyle (82). In terms of the role of GdpP in eDNA release in *S. aureus* biofilms, a mutation in *gdpP* would result in an increase in c-di-AMP levels, that would trigger the switch away from a biofilm lifestyle, and reduce expression of factors in *S. aureus* that promote cell lysis and release of eDNA (78). SarA has been previously shown to cause overproduction of extracellular nucleases that degrade eDNA (83), which would explain why a *sarA* mutant has more eDNA in its biofilms compared to wildtype. XdrA is a transcription factor and when mutated, was shown to increase expression of genes involved in cell envelope synthesis and stress response, which could contribute to increased cell envelope stability, reduced lysis and thus less eDNA release (78). Apt is a predicted adenine phosphoribosyltransferase, which is involved in nucleotide salvage, and when mutated would enhance *de novo* purine nucleotide synthesis, potentially preventing the drop in c-di-AMP levels and the shift towards a biofilm lifestyle (78). Overall, this study identified and characterized several novel factors involved in *S. aureus* biofilm formation with a particular focus on factors involved in eDNA release. In terms of genes in the “dark matter” category, there was one gene that is now predicted to have a role in eDNA release in *S. aureus* from the TIS outputs of this study (Table 1).

As mentioned at the start of this section, many biofilms are polymicrobial and thus, interactions between biofilm inhabitants can impact biofilm development and the host/local environment. Ibberson *et al.* (84) used TIS to understand how the essential genome of *S. aureus* differs between *in vivo* mono- and co-infection with *P. aeruginosa* in a murine chronic wound biofilm model. Compared to *S. aureus* mono-infection, many genes (182, representing 6% of the *S. aureus* genome) were uniquely essential for co-infection. They defined these as “community-dependent essential” (CoDE) genes. These fell into the functional categories of energy production and conversion as well as those involving amino acid, nucleotide, carbohydrate, lipid and inorganic ion metabolism, which suggests that *P. aeruginosa* may induce metabolic stress on *S. aureus* in this setting. Several noteworthy CoDE genes included a global regulator repressor of toxins, Rot, that represses expression of genes encoding toxins and proteases (which in turn reduces biofilm formation (85, 86)); the phenol-soluble modulin psmβ1, which is part of a toxin family that causes lysis of blood cells, stimulates host inflammatory responses, and contributes to biofilm development and dissemination of biofilm associated infections (87); and multiple components of the T7SS, which secretes effectors into host cells, causing cytotoxicity and immune modulation or into bacterial cells causing death/growth inhibition (88). Overall, these results suggested that *P. aeruginosa* co-infection is significantly modulating *S. aureus* virulence *in vivo*. To determine if the concept of CoDE genes was specific to *S. aureus*/*P. aeruginosa* and the chronic wound biofilm model, the authors performed TIS on the oral pathogen *Aggregatibacter actinomycetemcomitans* when in mono- and co-infection with the oral commensal *Streptococcus gordonii* in a murine abscess infection model (84). This revealed that 155 genes (∼47%) of the *in vivo* essential genome of *A. actinomycetemcomitans* were CoDE genes. As for *S. aureus,* the genes required for co-infection encoded multiple nutrient transporters and biosynthesis pathways, which suggests that *S. gordonii* induces metabolic stress on *A. actinomycetemcomitans* during co-infection. This study identified 1000’s of genes in total that were essential for *S. aureus* or *A. actinomycetemcomitans* under a range of settings including *in vitro,* and in several *in vivo-*like models, either alone or in the presence of another bacteria (84). In terms of shedding light on potential gene function ∼1680 of these genes are hypotheticals, which means that this study has been able to assign a predicted function to a huge number of genes that were previously considered to be genomic “dark matter” (Table 1). Overall, the findings from this study are particularly significant as the authors identified essential genes that are important for bacterial survival under conditions highly relevant to *in vivo* infection conditions. In addition to this, the TIS outputs include information on the essential genes for survival both in a mono- and polymicrobial biofilm. It would be interesting to expand the concept of CoDE genes to more bacterial species in relevant models, as this would provide a fantastic platform for beginning to understand how other important species survive in biofilm communities *in vivo.* It would also be informative to not only focus on essential genes but also on genes that provide a (conditional) *in vivo* survival advantage in mono-vs. co-infection.

Overall, these TIS studies have not only increased our understanding of the mechanisms involved in biofilm development and bacterial interactions but also in identifying bacteria- and biofilm-specific factors that are key for these processes. In terms of increasing our understanding of genomic “dark matter” from these studies, 1000’s of genes and several pathways have now been assigned a predicted function in different aspects of biofilm development *in vitro* or *in vivo.* As seen for the studies in the AMR section of this review, studies that looked at more than one bacterial strain (53, 84) and/or more than one condition (84) were those that were able to predict the function of the highest number of “dark matter” genes. Thus, again, while it is important to use TIS approaches to understand survival of single species under less complex (laboratory) conditions, it is also very informative to extend and combine these experiments with conditions that are more reflective of the real world *e.g.* polymicrobial communities and in *vivo*-like models.

## Bacterial virulence, infection strategies, and host-pathogen interactions

Antimicrobial resistance and biofilm formation are two main issues when it comes to controlling bacterial infections. Yet an in-depth understanding of how pathogens cause disease is also necessary to develop ways to treat infections and improve patient outcomes. TIS approaches have been crucial in providing information about the genes encoding pathogen virulence factors and host-interaction partners. The definition of a virulence factor has proved divisive but for the purposes of this review it will be taken to include anything that would allow a pathogen to survive in a host as well as determinants that directly compromise the integrity of the host.

Enterohemorrhagic *E. coli* O157:H7 (EHEC) is one of the Shiga-like toxin-producing types of *E. coli* and is an important foodborne pathogen that causes food poisoning which can lead to serious systemic complications. Warr *et al.* (89) passaged a TIS library through the infant rabbit colon to identify genes required for growth of EHEC *in vivo*. The authors confirmed that the EHEC locus of enterocyte effacement (LEE)-encoded type III secretion system (T3SS) apparatus, known to be involved in pathogenicity (90), is required for growth *in vivo*. Of the 49 T3SS EHEC effectors, only a few were crucial for *in vivo* fitness, namely *tir* and two non-LEE encoded effectors (Nle), *nleA* and *espM1*. Tir and EspM1 are key in the initial step of infection when triggering pedestal formation, which occurs due to rearrangement of actin in the host cell cytoskeleton beneath the bacteria, while NleA is required to inhibit vesicle trafficking (91, 92, 93). This observation is an interesting validation of a more recent study in *Citrobacter rodentium*, in which it was shown that among the available 21 effectors only Tir, EspZ, and NleA were essential but that a network of effectors is required for host adaptation in certain contexts (94). This suggests that TIS could be used to further document the suite of effectors actually contributing to fitness in various infection contexts and/or conditions. Warr *et al.* (89) also identified several other factors that were involved in promoting EHEC resistance to host-derived stresses including *cvpA, tatABC, sufI,* and *envC*. Previous TIS studies have classified *cvpA* as important for colonization of other enteric pathogens but did not investigate its role further (95, 96). Here a *cvpA* mutant was found to be outcompeted by wildtype EHEC *in vivo* and was much more sensitive to bile salts compared to wildtype. CvpA is a predicted inner membrane protein with 4 to 5 transmembrane domains and is partially similar to the inner membrane component of the Major Facilitator Superfamily (MFS) of transporters, which suggests that CvpA may be involved in substrate export, potentially of bile salts (89). SufI is a substrate for the twin-arginine translocation (TAT) protein secretion system (97), encoded by *tatABC,* which transports folded protein substrates across the bacterial cytoplasmic membrane (98). In the periplasm, SufI localizes to the divisome and is thought to be involved in maintaining divisome assembly when the cell encounters stress (99). EnvC is a murein hydrolase that is required for cell division (100). Additional experiments in the current study with *sufI, tatABD* and *envC* deletion mutants showed clear cell division and cell morphology defects in high osmolarity media (reflective of intestinal conditions), which suggests that impairing cell division is likely to decrease fitness of EHEC in the intestine. Given that multiple components that control cell division and morphology are key for *in vivo* fitness suggests that one or more of these components could be a valid therapeutic target to combat EHEC infections. In terms of genomic ‘dark matter’, this study identified more than 200 genes that now have a predicted function in *in vivo* survival of EHEC (Table 1).

*Legionella pneumophila* is the causative agent of Legionnaires' disease which is characterized by severe pneumonia. This pathogen encodes more than 300 different effector proteins that are delivered into host cells by the Dot/Icm type IV secretion system (T4SS) during infection (101), far more than the few described above for the EHEC T3SS. Identifying and unravelling the involvement of T3SS effectors is already very challenging, thus it is not surprising that it has proved very difficult to assess the importance of individual T4SS effectors for virulence. Shames *et al.* (102) initially used an arrayed (*i.e.* 96-well plate) transposon mutant library of *L. pneumophila,* composed of ∼10,100 mutants, and identified within this library the mutants of known or putative T4SS effectors, as well as of the T4SS machinery. Mutants that do not express a flagellum and are thus capable of escaping detection by the host inflammasome, were also included as internal controls. Where possible the authors included two different transposon mutants for each gene so that fitness changes related to that gene could be validated independently. The final TIS pool contained selected mutants in a total of 528 genes. This was inoculated *via* an intranasal route into mice or into bone marrow-derived macrophages (BMDMs) from mice. As expected, TIS revealed a significant increase in the proportion of flagellin-deficient mutants in the output pool, since these mutants have a competitive advantage over wildtype due to their ability to escape flagellin-mediated activation of the inflammasome. This concept of immune evasion has been previously reported (103), including for meningitis-causing pathogens evading microglia- and astrocytes-dependent immunity in the brain (104). In addition to identifying T4SS machinery mutants, several effectors were found to be important for survival, including effectors known to be involved in intracellular replication (*mavN* and *adhA*). The authors also found that mutations in several uncharacterized putative T4SS effectors (*lpg2505* and *ravY*) resulted in decreased intracellular survival. Lpg2505 is encoded downstream of the effector-encoding gene *sidI*, the product of which is toxic to eukaryotic cells (101). In several cases *L. pneumophila* effectors have been shown to modulate the activity of other effector proteins after delivery of the proteins into the host. These are called metaeffectors, or an “effector of effectors”, for example the well characterized *L. pneumophila* metaeffector LubX, which is a E3 ubiquitin ligase that targets another translocated substrate, SidH, for degradation during intracellular replication (105). In the current study the authors demonstrated that Lpg2505 was indeed a metaeffector, which regulates SidI activity to prevent host damage that decreases *L. pneumophila* intracellular infection and virulence levels (102). Further investigation of RavY in this study confirmed that it was a T4SS effector and that *ravY* is highly conserved in *Legionella pneumophilia* strains. The authors also found that a mutant of the previously characterized T4SS effector, LegC4, had a significant fitness advantage in the lungs but not in the BMDMs, suggesting that LegC4 increases *L. pneumophila* clearance by the host immune system. These findings demonstrate that using TIS to assess a complex process like the T4SS requirement during infection, could lead to increased understanding and ability to fine tune the role and synergy of associated effectors in various infection contexts, both *in vivo* and *ex vivo*.

Group A *Streptococcus* (*Streptococcus pyogenes,* GAS) is a human pathogen that causes more than 500,000 deaths each year (106). GAS typically infects the throat and skin and causes millions of self-limiting infections each year but is also capable of causing life-threatening invasive infections such as necrotizing fasciitis and streptococcal toxic shock syndrome (106). Breton *et al.* (107) used TIS to identify the genes required for *in vivo* fitness in a murine model of skin and soft tissue infection. Output pools of mutants were harvested from the infection site at 12, 24 and 48 h post infection, revealing 0, 75 and 106 genes, respectively, associated with decreased fitness. There were 34 genes common to both the 24 h and 48 h time points. For the same time points, the authors identified 0 (12 h), 29 (24 h) and 107 (48 h) genes linked to increased *in vivo* fitness, with 10 genes common between the 24 and 48 h time points. In total there were 54 genes in the genomic “dark matter” category (Table 1). Of the “dark matter” genes, RS06890 and RS06895 (renamed *scfA* and *scfB,* for subcutaneous fitness, in this study) were predicted to encode hypothetical membrane spanning proteins of unknown function. Non-polar single and double gene mutants were generated and these mutants inoculated into the murine mouse skin model either alone, or together with wild type. All mutants had a lower *in vivo* bacterial burden when compared to wildtype, and when inoculated with wildtype were outcompeted. Dissemination of each mutant was also assessed by quantifying bacterial burden in the spleen, with significantly lower numbers being observed compared to wildtype. A previous study has shown that mutating homologues of *scfA* and *scfB* in *Streptococcus mutans* had reduced growth under acidic conditions, and increased biofilm formation (108), suggesting that these phenotypes are also important for GAS infection in the context of skin and soft tissue infection (107). Overall, this suggests that ScfA and ScfB are involved in GAS adaptation and fitness during skin infection, and potentially in dissemination to other parts of the host beyond the skin (107). The identification of these two genes is highly relevant for them being potential therapeutic targets since they appear key in the invasion process. The fact that their function was unknown prior to this study is a superb example of the power of TIS in combination with further validation to illuminate biological functions of genomic “dark matter.”

These selected studies exemplify how TIS has been able to provide rich information about the genes required for virulence of several important bacterial species, with more than 10,000 genes that were previously genomic “dark matter,” now having predicted functions (Table 1). In all cases, the TIS outputs were a fantastic starting point to then undertake detailed experiments to obtain an in-depth understanding of the biological functions of several key gene products and their role in infection. All studies used complex *in vivo* models and thus the predictions and demonstrations of gene function are likely to be reflective of a real infection setting.

## Bacterial cell physiology and the rules of life

TIS approaches can address questions relating to specific bacterial behaviors, but it can also be used to understand more global and fundamental aspects of bacterial cell physiology, genome structure and chromosome organization, as detailed in this section.

The positions of naturally occurring transposable elements have been used to help understand genome evolution, for example, studies in *Drosophila melanogaster* have analyzed the patterns of transposons across the genome and compared these to transcription start site positions and knowledge of genome compaction (109, 110). This showed a higher propensity for insertions around regions of active transcription (assumed to reflect more open DNA regions) but strong selection against functional disruption of coding regions. Analogous approaches have recently been used in the study of microbes, including work with the fungal pathogen, *Phytophtora sojae*, analyzing the patterns of exogenous transposon inserts. Similar findings were made with a high prevalence of inserts at transcription units and correlation between insertions and transcription level of the target loci (111). These studies illustrate how transposon insertion patterns can help inform genome function and evolution. TIS has also been used to explore how genome location can impact expression in bacteria. Scholz *et al.* (112) used a transposon to deliver a reporter gene to >140,000 locations in *E. coli* which revealed a more than 20-fold variation in expression from the same unit in different sites, with ribosomal operons being regions of highest transcription and mobile elements regions of lowest potential transcription. This information goes beyond simple gene function analysis and begins to incorporate the roles of higher order chromosome structure in controlling phenotypes. This information would be very informative in many aspects, such as genetic engineering of bacteria for production of valuable commodities, which would benefit from information about where in a chromosome introducing expression constructs is most likely to give desired outcomes.

Bacteria experience many different environments, and many species are able to adapt to grow in very different conditions, understanding how this is achieved is a major aim of microbiology. Multiple TIS experiments have been undertaken that aim to address this question, however in most cases just one or a few conditions are assayed, typically with a single bacterial species. Price *et al.* (113) however embarked on a massive undertaking to use TIS to understand how 32 diverse bacteria grow under more than 100 different conditions (Fig. 2*C*). The bacteria were genetically tractable, represented six different bacterial phyla and 23 different genera, and were a combination of aerobes (30 bacteria), strict anaerobe (1 bacteria) and strictly photosynthetic (1 bacteria). A randomly barcoded transposon mutant library (RB-Tn-Seq) (114) was generated for each bacteria, followed by TIS to generate genome-wide maps of transposon insertions. Here Price *et al.* (113) identified between 289 to 614 genes per bacterium that were predicted to be essential (based on their absence in the RB-Tn-Seq libraries) and tested growth of the 30 aerobic bacteria in the presence of 94 different carbon sources, 45 different nitrogen sources, and 34 to 55 stress-inducing compounds including antibiotics and metals. This allowed mutant phenotypes to be assigned to more than 10,000 unannotated/poorly annotated “dark matter” genes (Table 1). Manual combining of these genotype-phenotype associations with comparative sequence analysis allowed the authors to predict specific functions for many ABC transporters, catabolic enzymes, proteins with domains of unknown function (DUFs), and to identify putative novel DNA repair protein families. They also performed analyses to identify “specific” genes that contributed to fitness under only one/a small number of conditions, and “cofitness” patterns where multiple genes in a bacterium contributed to fitness across multiple conditions. This revealed that 52% of genes had conserved functional associations (fitting either “specific” or “cofitness” patterns), and of these a large proportion had conserved associations across genera (81%) and phyla (59%). An example of the insights obtained for “dark matter” genes predicted to encode proteins with DUFs is for the family UPF0126, which were found to be important for glycine utilization in 11 bacteria. Since UPF0126 is predicted to be a membrane protein, the authors predicted that these genes encode a glycine transporter. The authors were also able to use a similar approach to assign biological function to many other “dark matter” genes, *e.g.* those associated with thallium stress response and sulphate assimilation. Despite this massive effort, with huge amounts of data generated and analysis performed, the authors were only able to identify functional associations for potential orthologues of 12% of bacterial proteins that lacked detailed annotations. This highlights a major challenge in microbial genomics, which is that simply generating more genome sequences and TIS outputs will only advance our biological understanding of genomic “dark matter” so far. What needs to be done is to use additional experimental and computational approaches, in combination with genome sequencing and TIS, to truly begin to understand biological functions. This is discussed in more detail in the section “Perspective and outlook.”

As shown in the preceding example, defining the set of essential genes for an organism can help define the minimal functions needed for growth in each condition. As the ability to make mutant libraries has become easier, many studies have used TIS to study multiple strains of a species/genus to generate refined lists of the core of essential genes in different organisms. For example, a study with nine independent mutant libraries of *P. aeruginosa* grown in a range of conditions concluded 321 genes were truly essential and encoded determinants crucially required for growth (115). A similar study recently compared the list of essential genes across 13 mutant libraries representing five genera of Enterobacteriaceae and concluded 201 genes were essential across the collection representing a relatively small number of indispensable functions for all strains in this family (116). These kinds of studies provide a fantastic basis for future work to obtain a comprehensive understanding of the biology of not just one single species but of multiple strains and/or genera. These outputs could provide significant advancements in a range of areas such as in development of species/genera-specific antibacterial therapeutics, and in biotechnology applications.

In parallel to these “pan-genome” studies, TIS has been used to make important fundamental discoveries about individual bacterial species with non-conventional lifestyles. *Bdellovibrio bacteriovorus* is a predatory bacterium that can prey on a wide array of Gram-negative bacteria, which includes human pathogens (117). These bacteria make contact and attach to their prey using their flagella and T4P, respectively (118, 119). Within 10 to 20 min of attachment *B. bacteriovorus* invades the prey periplasm and remodels peptidoglycan to create a spherical bdelloplast (120). During this time *B. bacteriovorus* also secretes enzymes to degrade the host cytoplasmic membrane (120). *B. bacteriovorus* remains in this protected environment to degrade the host’s cytosolic proteins and nucleic acids, using these to facilitate its own replication, ultimately resulting in lysis of the prey cell to release several daughter cells (120). While the lifecycle of *B. bacteriovorus* is well understood, the mechanisms of how predation occurs and why certain bacteria are sensitive to predation is less well understood.

Duncan *et al.* (121) used TIS to understand the factors that are important for increased or decreased sensitivity of *V. cholerae* to predation by *B. bacteriovorus*. A high-density transposon mutant library of *V. cholerae* was generated and infected with *B. bacteriovorus*. The determinants that reduced fitness in *V. cholerae*, *i.e.* genes that encode components that provide resistance to predation, were flagellar motility genes, as well as genes involved in the generation of the cell envelope and LPS. This also included genes encoding factors involved in O-antigen biosynthesis and transport. The major genes that increased fitness in *Vibrio cholera*, *i.e.* genes that encode components that make the cell more sensitive to predation, included virulent regulators encoded by *toxR* and *toxS*. In terms of genes in the “dark matter” category from the TIS outputs in this study there were a total of 21 genes (Table 1), which were all predicted to encode factors that make the cell more resistant to predation. Follow up of clean deletion mutants of non-motile and non-flagellated (Δ*flrA*) or non-motile but flagellated (Δ*motY*) *V. cholerae* strains revealed that both mutants had decreased resistance to predation, which shows that a functional flagellum is required for a cell to be resistant to predation. This is suggested to be due to the increased fluctuations in drag force that occurs because of the tumbling that *V. cholerae* undergoes when swimming, which reduces *B. bacteriovorus’* ability to attach, and remain connected to the *V. cholerae* cell, to undergo predation. The authors also confirmed several other components that are required for predation resistance including OppC, the transmembrane component of the ABC transporter permease system for small peptides (122), and ManC, which is required for LPS O-antigen biosynthesis (123). While the role of OppC in resistance to predation is unclear, this result suggests that LPS is a key cellular component that might block predation. This study increased understanding of the factors that make *V. cholerae* sensitive and resistant to *B. bacteriovorus* predation as well as showing that many genes can have different roles depending upon the conditions.

TIS has been used extensively to increase our understanding of mechanisms that make bacteria resistant or sensitive to antibiotics, as detailed above in the section “AMR mechanisms and antimicrobial drug discovery.” *B. bacteriovorus* has been referred to as a living antibiotic (121) and while it is not in clinical use, it may be developed into a therapeutic option in the future. Key information for this development would be to know what makes prey cells either sensitive or resistant to predation. The study by Duncan *et al.* (121) supports the use of TIS to obtain the same type of information for other prey pathogens.

As described earlier, *L. pneumophila* is the causative agent of Legionnaires disease. This Gram-negative bacterium is ubiquitous in freshwater environments where it can acquire new genetic material *via* intra- and inter-kingdom horizontal gene transfer events in a process called natural transformation. This process is defined as the uptake of naked DNA into the cell from the environment *via* a specialized transport machinery, either a competence pseudopilus for Gram-positive bacteria or the T4P for Gram-negative bacteria, and recombination into the genome (124). In most species, the components required for natural transformation are not constitutively expressed and are instead regulated by the cell to induce a state called competence. In L. *pneumophila*, the core genes in the DNA uptake system are subjected to post-transcriptional repression by a ribonucleoprotein complex consisting of RocR and RocC (125). However, while there is some information about conditions that induce competence in *L. pneumophila,* this is generally poorly understood.

To understand when *Legionella* expresses machinery to facilitate DNA uptake Hardy *et al.* (126) used TIS to identify genes involved in natural transformation and competence induction. Natural transformation was followed by the addition of genomic DNA, which carried a kanamycin resistance cassette, facilitating selection of mutants capable of natural transformation by antibiotic selection. As expected, genes that were essential for this process (*i.e.* mutants absent after kanamycin treatment) included those encoding the T4P and other periplasmic- and inner membrane-associated natural transformation components. Genes not related to these components that had a partial defect in natural transformation included *djlA*, which encodes a DnaJ-like protein previously described as required for intracellular replication in *Legionella dumoffii* (127), and *lpp3030*, which encodes an uncharacterized protein with a putative signal peptide, suggesting an extra-cytoplasmic localization. The genes *letS* and *letA* were also hit in the TIS screen. LetS and LetA are the sensor and response regulator components of a two-component system (TCS), and homologous to the BarA/UvrY TCS in *E. coli* and GacS/GacA TCS in *P. aeruginosa*. Study of L. *pneumophila* clean deletion mutants of *letS* and *letA* revealed a huge reduction (>500 fold) in transformation levels. As mentioned earlier, the competence system in *L. pneumophila* is controlled by post transcriptional repression of the complex RocC/RocR, thus a *rocC* mutant is constitutively competent (125). The same reduction in transformability was not observed in a *rocC* and *letS/A* double mutant. This suggests that LetS/LetA are involved in regulation of competence upstream of the post transcriptional repression RocC/RocR pathway, which increases understanding of competence regulation in *L. pneumophila*. Two putative pilin homologs were also identified in the TIS screen, namely *pilA2* and *pilE.* Clean deletion mutants of these also had a defect in natural transformation. Follow up work suggests that PilA2 is the major T4P monomer subunit, since it was observed to assemble into long extracellular filaments. In a *pilE* mutant, there was a decrease in numbers of assembled T4P filaments, which suggests that PilE is important for pilus formation. Since one proposed role of minor pilins has been to localize to the T4P tip and stabilize the filament (128), the outputs from this study suggests that this is the role of PilE in *L. pneumophila*. Additionally, since some T4P assembly still occurs in a *pilE* mutant but that natural transformation is abolished, suggests that PilE is the DNA receptor at the external tip of the T4P in *L. pneumophila*. There was also a strong transformation defect in three genes, *lpp1976-lpp1977-lpp1978*. A mutant lacking all three genes also had a reduction in T4P number and length. Lpp1977 has homology to the putative minor pilin, PilW, with all three predicted proteins possessing an N-terminal transmembrane segment, and Lpp1977 and Lpp1978 both having a signal peptide, to designate these proteins for transport out of the cytoplasm. These features as well as the operon structure is like the characterized T4P minor pilin genes in *Neisseria meningitidis* and *P. aeruginosa.* Overall, this suggests that these genes may encode a set of minor pilins, which serve as the initiation complex for assembly of the T4P. In addition to these genes there were 13 genes in the “dark matter” category that are now predicted to have a role in natural transformation of *L. pneumophila* (Table 1). This study is a good example of how TIS provided a basis to increase knowledge on an important biological process possessed by this pathogen. In most cases the genes identified were already known to be involved in natural transformation, but the TIS output provided clues for how these genes could intersect with genomic “dark matter” components, which then directed further experimental work to increase understanding of how the transformation machinery is assembled and how competence is regulated.

The examples above show how TIS can efficiently predict roles for genes in varied situations. These studies are excellent examples of how TIS data can provide functional predictions for “dark matter” genes but also predict novel roles for already characterized genes in regulatory pathways and/or cellular processes. These studies also demonstrate how TIS outputs provide a fantastic basis for hypothesis development and further experiments to increase biological understanding. As researchers study more diverse growth conditions, incorporating fitness data for loci from TIS experiments with genome annotations will expand the available database of information that can be used to link genotypes to phenotypes.

## The use of TIS approaches in complex scenarios

**Figure 3:**
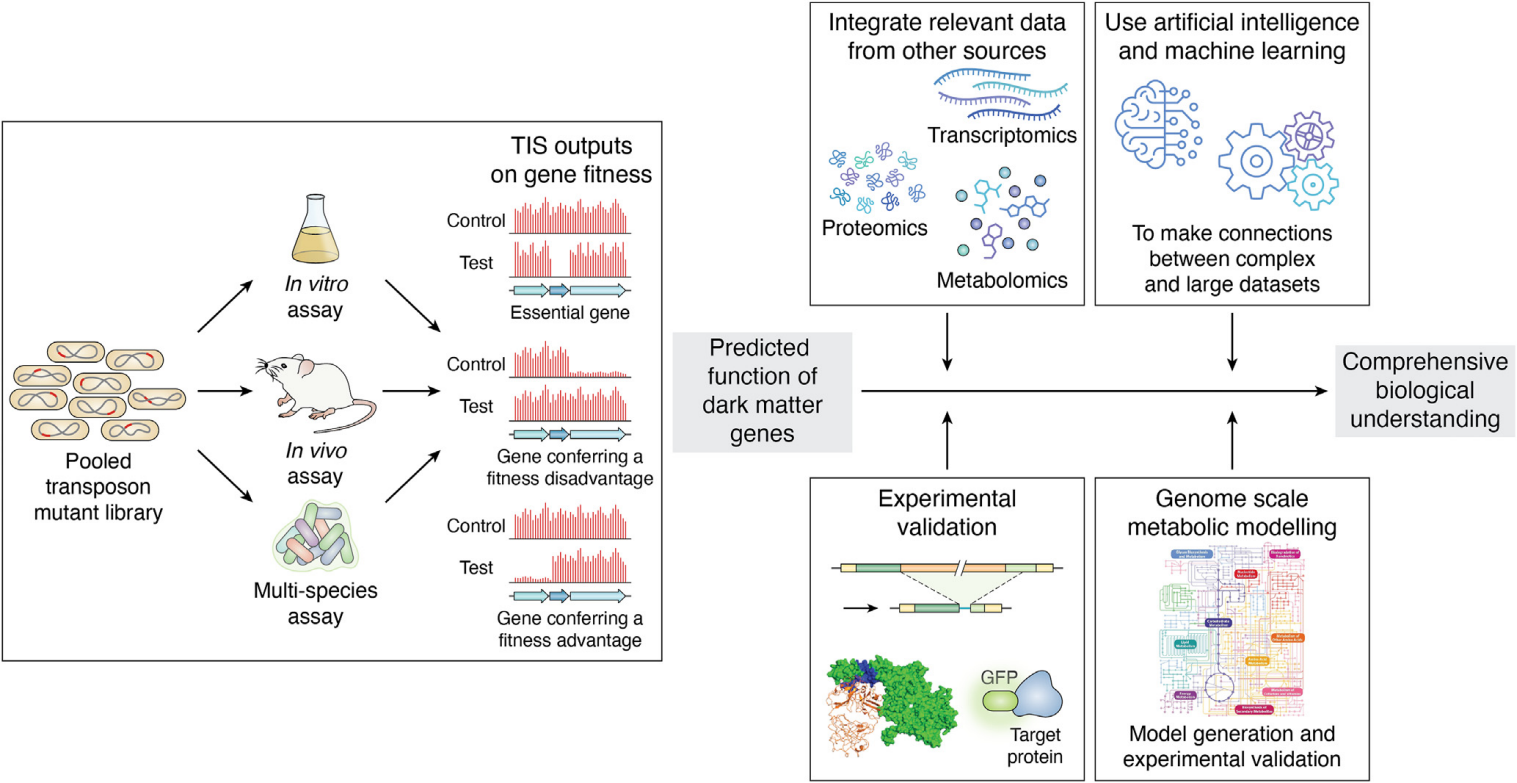
**Building upon TIS outputs to go from a predicted function of “dark matter” genes to a comprehensive biological understanding.** TIS approaches can be conducted in any number of assays, from *in vitro* to *in vivo,* and with single or multi-species communities. All these experiments can provide predictions about the function of genes in the “dark matter” category. Integration of these data with data generated in other approaches is essential to obtain true biological understanding. For example, to combine TIS outputs with outputs from 'omics datasets (*e.g.* transcriptomics, proteomics, and/or metabolomics) that were conducted under the same conditions; to validate the predictions using genome scale metabolic models from TIS outputs and experimentally validate these models; to use artificial intelligence (AI) and machine learning (ML) approaches to make structure-function predictions (*e.g.* AlphaFold3), to mine TIS datasets and make connections between TIS outputs for similar phenotypes (*e.g.* AMR, biofilms development, infection models) for different bacteria, and for future and retrospective TIS datasets.

Whilst there are numerous examples above showing how TIS can predict function of gene products from experiments performed with high-density transposon mutant libraries alone, the detail this provides is mainly predictive and requires additional work to obtain an in-depth knowledge of biological function. One approach that can increase the level of information obtained about the functional prediction can be to combine TIS with outputs from other datasets (Fig. 3). For example, TIS data that provide information about the importance of individual genes or pathways in defined conditions has been used in conjunction with genome-scale metabolic models (GSMM) (129, 130, 131, 132). These models correlate genomic information with physiological information for a bacterium, under defined conditions, to construct a metabolic network model. This model is refined, then tested and debugged through experimentation. To facilitate the linkage of TIS data with GSMMs specific tools have been made including “Tn-Core,” which helps relate the two approaches to refine identification of essential metabolism in a given condition (130).

One example of combining TIS with GSMMs, and in this case also with measurements of growth, metabolite utilization, and gene expression, is with an isolate of *S. pyogenes* serotype M1 (133). *S. pyogenes* causes over half a million deaths per year, with strains of the M1 serotype being widespread and those most commonly used to study virulence mechanisms. Inclusion of TIS data into an initial GSMM allowed the accuracy of predicted gene essentiality to rise to over 92% (from a baseline of 74% in the initial model). Comparison of the GSMM with TIS data about gene essentiality was able to identify previously unknown metabolic capabilities that must exist in *S. pyogenes*. For example, the nicotinamidase (NNAM) pathway was predicted to be essential by the model, but empirical TIS data showed it was not. This suggested the existence of an alternative nicotinate synthesis pathway in *S. pyogenes*. Reanalysis of metabolic pathways where genes became essential in selected growth conditions allowed the L-aspartate oxidase (ASPO6) and quinolinate synthase (QULNS) reactions to be identified as essential and were incorporated into the model to circumvent the original pathway. This is an example of TIS being able to identify new functions for genes, as well as illuminating a previously “dark matter” pathway. This improved model can now be used as a tool to predict what *S. pyogenes* requires to survive in a given condition, with the aim being that this can be exploited to develop novel therapeutics targeting previously unappreciated essential genes.

Another example is the combination of TIS data with phenotypic Biolog Microarrays, *in vivo* transcriptome data, and a GSMM of a hypervirulent *A. baumannii* clone, AB5075, which was used to determine how this organism survives in a murine bloodstream infection model (131). *A. baumannii* is a leading cause of septicemia in patients in the intensive care unit (ICU), which is associated with high levels of mortality (134). Combining TIS data with the draft GSMM for AB5075 allowed new functions to be assigned to “dark matter” genes. Comparisons of predicted essentiality of reactions with Biolog growth data and TIS data identified mismatches. For example, the draft GSMM did not predict a capability for AB5075 to grow with glycyl-l-asparagine or l-alanyl-l-histidine as sole nitrogen sources, however comparing TIS data for essential genes in these conditions with homology searches of hypothetical genes identified two previously unknown aminopeptidases (ABUW_2837 and ABUW_3646) whose incorporation into the GSMM resolved the inaccurate growth prediction. After using TIS data to help refine the model, transcriptomic data from *A. baumannii* AB5075 in a murine infection model were captured 2 and 4 h post-infection. This found 1408 genes differentially expressed at 4 h post-infection compared to 2 h. Of these, 396 genes were mapped to the GSMM, which identified 198 reactions with significantly altered fluxes between the two time points. Amongst the pathways with the most significantly increased changes were large increases in flux within peptidoglycan and LPS biosynthesis, indicating these are crucial in *A. baumannii* for growth in the bloodstream and/or establishment of bacteremia. This is a clear demonstration of how combining TIS data with data from other approaches can provide a significant advancement in biological understanding (Fig. 3).

A similar approach was also recently used to predict mutations that impact production of polyhydroxybutyrate by *Cupriavidus necator*, which is being developed as a biotechnology chassis bacterium (132). *C. necator* has become an organism of interest in biotechnology due to its resilience, ability to produce a range of platform chemicals using CO_2_ as a starting material, and genetic tractability. In this study TIS was used to inform and refine a GSMM which could reliably predict essential genes in different growth conditions. Over a million transposon mutants were made and TIS data from various growth conditions were compared with predictions from GSMM. This showed that the GSMM was able to predict essential genes with good accuracy (81% of predicted essential genes were scored as such in the TIS data) but the recall of the model was poorer, with only 61% of genes called essential by TIS being predicted to be so by the GSMM. The authors suggest various reasons for this relatively low rate of recall including a lack of regulation data and incomplete annotations in the model. Importantly, recall was improved to 69% when transcriptomic data was also used. This work again highlights the power of combining TIS with other data sets to increase understanding but also identifies some of the unresolved challenges in integrating TIS with GSMMs. Additionally, this study suggests routes to engineer *C. necator* for industrial applications to convert greenhouse gases into biochemicals and biofuels (132).

As well as combining datasets, TIS is also now being applied to more complex environments. Microbes rarely live in isolation, and growth in a community context can have large impacts on the genes an individual needs to survive, for example, a metabolic requirement may be conferred by a neighboring cell expressing genes that are essential in a polymicrobial setting but are redundant when that microbe is in isolation. For example, a study of how EHEC responds to a panel of commensal gut isolates, known to provide competitive exclusion against other pathogens, combined data from RNA-seq and TIS (135). This identified two major responses in EHEC when it was co-cultured compared to growth alone: repression of biotin production, which was correlated with high levels of production by the microbiota, making *de novo* production by EHEC unnecessary, and increased expression of oxidative stress responses. For the latter, the *uhpA* gene became very important when in co-culture. Follow-up experiments demonstrated that UhpA was needed to oxidize excess NADPH that accumulates when EHEC takes up and metabolizes microbiota-derived acetate (135), since balancing cellular NADPH levels is crucial for a cell to effectively respond to oxidative stress (136).

Being able to target specific organisms or genes within a microbial community would allow precise engineering of complex communities and allow targeted manipulation to modify function and/or outputs. A recent study (137) has shown this to be possible, where *in situ* communities were randomly mutagenized with transposons (using a method called environmental transformation sequencing, ET-seq). Following introduction of transposomes (a complex of proteins and DNA that is involved in DNA transposition) into the target community (by electroporation or conjugation), the method combines metagenomics to quantify the diversity and abundance of members within the community, alongside enrichment and sequencing of transposon–host insertion junctions. Together, the two methods allow quantitative measurement of transformation efficiency for different species within the microbiota. After establishing which members of a community are accessible to introduction of exogenous DNA, the authors developed a follow up method to specifically edit these targets within the community called “DART” (DNA-editing all-in-one RNA-guided CRISPR–Cas transposase). DART uses an RNA-guided CRISPR–Cas Tn7 transposase that delivers desired DNA encoded within transposon mosaic ends into a specific location of a target genome defined by a guide RNA (encoded on a plasmid alongside the cargo DNA). The whole approach was validated with a synthetic community of nine species isolated from soil where five genetically accessible species were identified and targeted editing was demonstrated to be feasible (137). Subsequently the method was applied to a more complex community from an infant stool sample and specific delivery of antibiotic resistance cassettes to two different genomic locations in separate *E. coli* strains was demonstrated. This work provides a potentially revolutionary platform for a new generation of experiments to study and modify microbial communities *in situ* and to understand the function of any “dark matter” in the strains which prove amenable to targeting (Fig. 3).

Using the base data from TIS experiments in conjunction with other analytical approaches can be hugely valuable. One example of this is the combination of TIS with ML. Santiago *et al.* (138) used TIS with ML to identify the mode of action and mechanisms of resistance of *S. aureus* to 32 different antibiotics. These antibiotics all had known mechanisms of action, and all major targets for broad-spectrum antibiotics were represented. The authors were able to use this data as a training set to both identify known targets and the mode of action for many of these antibiotics but also to predict novel mechanisms of action for new drugs. A key example of this is the mode of action of the lysocins, which are a family of bacterolytic lipodepsipeptides. The author’s ML approach was able to predict that the cell wall precursor Lipid II was the molecular target of lyocins in *S. aureus*, which they then went on to validate experimentally (138). This kind of approach has paved the way for combining TIS with ML or other artificial intelligence (AI)-based approaches and will be likely to assist with tackling TIS data mining that has come from more complex environments and microbial communities (Fig. 3). As technical capabilities pave the way for generation of very large datasets it will become crucial to develop analytics that can derive patterns from much larger series of experiments than in conventional TIS studies.

## Perspective and outlook

TIS approaches have proved to be a fantastic tool for predicting the biological function of genes in the “dark matter” category. From the studies detailed in this review, there are now >10,000s genes, which were previously hypothetical, that now have a predicted function from TIS outputs (Table 1). These studies also highlight the power of TIS in assigning new predicted roles for genes that were already linked to an existing phenotype. In many cases, these TIS predictions formed the basis for further experimental work to obtain a detailed insight into biological function. This latter, often labor-intensive step, is essential to go beyond a list of genes with predicted phenotypes to actual experimental validation. Indeed, TIS studies that include detailed experimental validation are often the ones that provide the greatest amount of insight into biological function. Similarly, higher levels of insight were obtained from TIS studies that were *in vivo* and/or models that are close to the real-world conditions; that used relevant mixed bacterial communities instead of a single species; and that undertook TIS experiments with multiple strains, antibiotics, and/or conditions. Future studies should consider these aspects when designing their experimental setup to maximize the impact and utility of TIS outputs.

Since the time and effort required for experimental validation of TIS outputs is often extensive, it is of course important to pick the “best” genes to follow-up. Typically, researchers will pick genes that have the “strongest” TIS score (*i.e.* hits that are significant and have the greatest fold change compared to the control) and have some predicted function/conserved motif that could link the gene to the phenotype of interest, since this provides a more obvious experimental pathway to validation. Genes that fall into the true “dark matter” category, having no known protein sequence or predicted structural homologs, nor any conserved domains, are generally not selected for follow up experiments as the next steps for experimental validation are less clear. However, this approach of studying genes that are similar to what is already known means that we are often only making small, incremental steps in understanding. This limits our ability to truly expand and accelerate biological understanding and is blunting the power of TIS approaches.

As shown from the studies in this review, there are now 10,000’s of hypothetical genes that have predicted functions; however, only a few of these genes have been followed up to experimentally demonstrate biological function. This means that while these genes are no longer completely “dark matter,” *i.e.* we have a prediction of their biological function, we still do not have a deep understanding of their role within the microbial cell. How can we address this? Efforts have been made to increase the throughput of extracting transposon insert abundance with highly parallelized automated identification of significant hits. One key example is AlbaTraDIS (139), which can process large numbers of samples in parallel and generate networks representing relationships between datasets to help understand relationships between microbial responses to diverse conditions. Additionally, comparisons of multiple TIS libraries of a single species under diverse conditions have also been recently described (115). Therefore, our ability to extract data from a large series of TIS experiments is beginning to be realized. However, these additional analyses have been restricted to relatively small samples with single mutant libraries or single species, and it remains challenging to extract meaningful information from larger, more complex datasets. One future direction to address this could be to harness the power of AI to mine TIS datasets, and to make connections not just within a single dataset, but between TIS datasets for the same bacteria and/or for different bacteria but on the same biological function *e.g.* resistance to the same antibiotic, biofilm development, infection of the same *in vivo* model. It would also be informative to interrogate these outputs against other available datasets such as those from transcriptomics, metabolomics and proteomics studies, and to also incorporate information from AI tools like AlphaFold3 (140) (Fig. 3). All these additional levels of analysis could be utilized not just for future TIS datasets but also performed retrospectively. It is likely that we are only at the tip of the iceberg in terms of data utilization and generation of biological insight from TIS studies, given that these approaches have been used for more than 15 years and generated huge numbers of datasets. For more details on how AI methods could be used for big datasets, see Zhe *et al.* (141) where they review the use of AI in probing microbial dark matter in microbiome datasets.

Whilst modern TIS experiments are based on assaying large pools of mutants in parallel, there are some disadvantages compared to classic transposon screens, where each mutant was assayed individually. In the example in the biofilm development section above, where gene products are involved in public good production, their involvement can be masked due to the complementation of any impact from neighbors. This shows how some resolution of assigning genotype-phenotype can be lost in an approach that utilizes mutant pools. Recent developments in droplet genomics and single-cell analysis (*e.g.* droplet Tn-seq (142)) now allow the potential for very large numbers of transposon mutants to be assayed in a high-throughput manner while still allowing separation of individual mutant(s) into spots/pools. This will not be suitable for all conditions but offers an interesting route to providing unambiguous linkages between genotypes and phenotypes and promises to close the gap on a lot of genomic “dark matter.”

An additional challenge with TIS experiments is that standard TIS approaches are unable to study the function of genes that are essential for the phenotype being assayed. One way to address this is by using gain of function insertions with transposons that contain outward-facing inducible promoters, which allows the neighboring gene to be repressed (under uninducing conditions) or overexpressed (under inducing conditions). The TIS experiment can be performed in both conditions and changes in transposon insertion frequency compared, which can allow identification of essential genes since their activity is dependent upon the promoter activity. We have developed TraDIS-Xpress, where we used a transposon (Tn5) with an arabinose inducible p_BAD_ outward-facing promoter and obtained transposon insertion data under both inducing and uninducing conditions for *E. coli* in the presence of triclosan (142). We developed the TraDIS-Xpress software package which was then able to identify both essential and non-essential genes for *E. coli* triclosan tolerance (143). Outward-facing promoters have also been used to identify essential genes in other bacteria such as *S. aureus* and *Caulobacter crescentus* (22, 144, 145). Some studies have also used constitutive promoters with different strengths, which can also identify essential genes (22, 145). These strategies that facilitate the study of essential genes in a high throughput manner further increases the power of TIS.

Whilst TIS approaches have been applied to many important and well-studied species there are still microbes where the tools have not yet been developed to allow large scale generation of transposon mutant libraries. Indeed, the inability to perform TIS with microbes that are not genetically manipulable, that are multidrug resistant (since antimicrobial resistance is typically used to select for transposon-containing mutants), or that do not grow well under laboratory conditions has really limited the amount of diverse biological insight being obtained. These challenges are slowly being overcome as we develop the ability to grow and genetically manipulate more and more species, including less well-studied bacteria, representing more microbial diversity. For example, TIS has been recently applied to *Saccharibacteria*, a member of the candidate phyla radiation, which represents a significant but under-explored part of microbial life (146). These bacteria are very small (100–200 nm wide) with tiny genomes (<1 Mb) and limited predicted metabolic capacity, and thus, it was assumed that these bacteria require a host organism to support their growth (147). Indeed, it was recently shown that the growth of *Saccharibacteria* relies on co-cultivation with the host bacteria belonging to the phylum Actinomycetota (148). TIS of *Saccharibacteria* was achieved by exploiting the natural competence of this bacterium and also by co-culturing with the host bacterium *Actinomyces israelii* (146). This work revealed functions for many enigmatic genes, such as those involved in *Saccharibacteria*-host interactions and in the production of macromolecular complexes (T4SS, T4P, and adhesins) and biofilms (146). Continuing to develop genetic tools in previously recalcitrant organisms will provide obvious rewards and will further help decipher the vast “dark matter” of microbial genomes beyond model organisms.

The development and application of TIS as a tool to predict biological phenotype from genotype has been foundational to much of contemporary microbiology, with these predictions providing a fantastic basis to direct experimental work to obtain an in-depth understanding of biological function. Looking forward, there is an urgent need to develop tools to link TIS outputs with other relevant datasets and ways to deal with these large datasets to extract relevant biological meaning. This offers an exciting challenge which, if achieved, will accelerate the production of more groundbreaking discoveries in microbiology.

## Data availability

All data described are in the article main text.

## Conflict of interest

The authors declare that they have no conflicts of interest with the contents of this article.
